# Translation, adaptation, and validation of the Self-efficacy in Palliative Care scale (SEPC) for use in Swedish healthcare settings

**DOI:** 10.1186/s12904-022-00940-5

**Published:** 2022-04-11

**Authors:** Lisa Granat, Sofia Andersson, Emina Hadziabdic, Margareta Brännström, Anna Sandgren

**Affiliations:** 1 Center for Collaborative Palliative Care, Department of Health and Caring Sciences, Linnaeus University, 351 95 Växjö, Sweden; 2 Department of Nursing, Umeå University, Campus Skellefteå, 901 87 Umeå, Sweden

**Keywords:** Confidence, Healthcare professionals, Instrument, Palliative care, Self-efficacy, SEPC-scale, Validation

## Abstract

**Background:**

One challenge for healthcare professionals when delivering palliative care can be their lack of confidence. The Self-efficacy in Palliative Care Scale (SEPC) is considered a valid and reliable assessment scale to evaluate confidence when delivering palliative care. Currently, there is not a reliable instrument aimed to measure healthcare professionals’ confidence in palliative care in Swedish. Therefore, this study aimed to translate, culturally adapt, and validate the SEPC-scale for use in a Swedish healthcare context.

**Methods:**

This study applied the World Health Organization’s (WHO) guidelines for translating and adapting instruments, using forward and back-translation, an expert panel, and cognitive interviews. Swedish experts in palliative care (*n* = 6) assessed the Swedish version of the SEPC-scale based on its relevance, understandability, clarity, and sensitivity on a Likert scale. Methods involved calculation of content validity index (CVI) with modified kappa statistics and cognitive interviewing with healthcare professionals (*n* = 10) according to the “think-aloud” method.

**Results:**

Calculation of I-CVI (Item-CVI) showed that the Swedish SEPC-scale was considered relevant but needed some modifications to improve its understandability and clarity. The experts recognized an absence of precision in some items that affected clarity and understanding. Likewise, the healthcare professionals highlighted some challenges with understandability and clarity. They indicated that the scale was relevant, but a few items needed adjustment to fit a broader range of healthcare professionals. Items that referred to death and dying could be sensitive but were considered relevant.

**Conclusions:**

The SEPC-scale is considered valid for use in Swedish healthcare practice, for a broad range of healthcare professionals, and for diagnoses other than cancer. This study shows that cultural adaptation is necessary for establishing relevance and enabling acceptance to various healthcare professionals and contexts in the target country.

## Background

Globally, there is an increasing need for palliative care [1], particularly owing to the situations created by the COVID-19 pandemic [2, 3]. The coronavirus has affected the older and most fragile population the most due to physical comorbidities and greater mortality [4], and has accentuated the importance of a health care system that can deliver palliative care [2, 3]. Despite this knowledge, healthcare settings have struggled to achieve this goal [5, 6]. The World Health Organization (WHO) stipulates that “ Palliative care is an approach that improves the quality of life of patients (adults and children) and their families who are facing problems associated with life-threatening illness. It prevents and relieves suffering through the early identification, correct assessment and treatment of pain and other problems, whether physical, psychosocial or spiritual” [1]. Palliative care has a holistic view [7]. Thus, in order to provide high-quality care, healthcare professionals ought to have the ability and core competency to meet patients’ and families’ physical, social, psychological, and spiritual needs. Healthcare professionals include but are not limited to nurses, physicians, psychologists, physiotherapists, social workers, and occupational therapists [8]. Today, a particular challenge for healthcare stems from equipping healthcare professionals with the skills and competencies necessary to provide palliative care. Many healthcare professionals find it arduous to deliver this type of care because they have little experience or awareness about the palliative approach [9]. This shapes the behavior of healthcare professionals who are unprepared to care for patients with palliative needs, creating unsatisfactory situations for patients and their families [10].

The foundation for improving palliative care is the opportunities that healthcare professionals have to acquire training and education [5, 11]. In this context, the theory of self-efficacy can constitute a useful instrument in both practice development and healthcare research. The theory of self-efficacy is a social cognitive theory based on the work of Albert Bandura, who articulates that human competence and capability links to action and practice [12]. Bandura argues that people’s beliefs about their capacity and capability (perceived self-efficacy) will affect the actual outcome of performing a specific behavior or skill. People with low self-efficacy will mostly avoid tasks in particular areas as they consider them difficult or believe they are unlikely to succeed, resulting in low intrinsic motivation and anxiety/fear. Correspondingly, people with strong self-efficacy are more motivated to complete tasks; they are unafraid, interested, and will adapt to find new or alternate ways to reach their goals. Personal self-efficacy is not purely affected by personal determiners; external behavior determiners, such as the contextual surroundings in organizations and the social environment, influence a person's ability to perform [12]. Thus, self-efficacy is not a measure of someone’s capability; it is a measure of what a person thinks they can perform in specific areas [12]. As a research tool, self-efficacy could be useful to evaluate healthcare professionals’ confidence and preparedness to deliver palliative care and can be considered a helpful tool in planning and evaluating quality improvements in clinical settings and in education and training [13]. In healthcare in general, as well as in palliative care, studies have shown that a high level of perceived self-efficacy causes healthcare staff to feel more comfortable and deliver better care [14–17].

The Self-efficacy in Palliative Care scale (SEPC) was developed by Mason and Ellershaw [18] with the intention of assessing the outcome of a palliative care education program at Liverpool University. In a pilot test, medical undergraduates (*n* = 139) completed the scale before and after completion of a two-week education program. The SEPC-scale, developed in line with Bandura’s theory of self-efficacy, consists of three theoretical subscales that assess perceived self-efficacy in communication, patient (symptom) management, and multidisciplinary teamwork. Mason and Ellershaw’s initial study results showed that the SEPC-scale is valid and a reliable assessment scale; with a Cronbach’s α value greater than 0.92. Furthermore, three factors occurred during the factor analysis that established the item distribution in the subscales and declared over 68% of the variance [18]. Other countries have also found the scale to be useful. For example, both a Spanish version and a Brazilian Portuguese version were translated and adapted for their specific cultures, with results identifying that the instrument is valid for use in these countries [19, 20]. A multicenter study, including 6 European countries (Belgium, England, Finland, Italy, the Netherlands, and Poland) used the SEPC communication subscale as a measurement for nurses’ and assistant nurses’ self-efficacy towards end-of-life communication. However, it remains unclear whether this has achieved a complete validation of the instrument regarding a cultural adaptation to the target country [21]. Currently, there are no reliable and valid instruments available in Swedish which measure healthcare professionals’ (such as registered nurses, assisted nurses and physicians) beliefs about their ability to provide palliative care. The SEPC-scale could be used in specific educational programs and /interventions and for continuous education. Having access to such an instrument provides an opportunity to develop a baseline estimation before training, examine effects during and after an educational intervention, or for continuous training, and evaluate quality improvements in clinical settings. The SEPC-scale can be used as a comprehensive instrument because it includes three fundamental features of palliative care: communication, patient management, and multi-professional teamwork. However, the instrument focuses on cancer diagnoses and medical professionals’ self-efficacy [18]. The aim of this study is to supply Swedish care settings with a broad instrument aimed at all healthcare professionals who care for groups suffering from a serious illness; therefore, the instrument should be useable in multiple care settings. The data from a comprehensive instrument can provide a deeper understanding of whether Swedish healthcare professionals are ready to practice palliative care, the area in which they feel they need more training, and the areas in which they are more comfortable. Therefore, the aim of this study was to translate, culturally adapt, and validate the Self-efficacy in Palliative Care scale (SEPC) for use in the Swedish context.

## Methods

### Instrument

The SEPC-scale [18] consists of three subscales and a total of 23-items, asking about perceived self-efficacy in delivering palliative care. Every subscale has several items, which represent several behaviors and skills in the domain of palliative care: Communication (8 items), Patient management (8 items), and Multidisciplinary teamwork (7 items).

In the SEPC-scale, participants are asked to rate their confidence in their ability (perceived self-efficacy) to successfully perform each behavior or skill on a visual analog scale (VAS). The scale ranges from very anxious to very confident with a score range between 0–100. Higher scores suggest a higher confidence for each item, and a lower score shows a lower degree of confidence in performing the task.

The research group received permission from Dr. Stephen Mason, one of the original developers of the SEPC-scale, to translate and culturally adapt the instrument to the Swedish context.

### The process of translation, adaptation, and validation

This study sought to engage in a thorough translation and cultural adaptation, focusing on the instrument’s cross-cultural and conceptual aspects. The methodological approach followed World Health Organization’s (WHO) guidelines for translating and adapting instruments. The method is designed to enable the instrument to be usable and reach the same accepted status in the target country as it has in the country of origin [22].

For the SEPC-scale to be usable and accepted in the Swedish context, it is essential to validate the translation. This study used content validation and face-validation. Used together, these methods examine essential aspects of whether the items clearly address the proposed subject matter and whether the range of aspects is adequately included. Content validation explores the extent to which the set of items covers different components of interests in the instrument [23]. Face-validation examines the appropriateness, sensibility, or relevance of the sets of items in the instrument as they appear to the individuals completing the test. More formally, face-validation examines the degree to which test participants assess the content as relevant to the context in which the test is to be administrated [24]. According to the WHO, the instrument should be evaluated based on whether it is understandable, clear, or offensive [22]. The SEPC- scale was evaluated based on its relevance, understandability, clarity, and sensitivity to cover all vital translation and validation aspects. Content-validation and face-validation were obtained through experts in palliative care and cognitive interviews with healthcare professionals. The study was performed in four steps: Step 1. Forward-translation and back-translation, Step 2. Expert panel review, Step 3. Cognitive interviewing, and Step 4. Final version.

#### Step 1: forward-translation and back-translation

One independent translator whose mother tongue was Swedish did the translation from English to Swedish (forward-translation). Additionally, each member of the research group (*n* = 4) did their own independent translations. The research group compared all five translated, independent versions of the SEPC-scale and searched for the most appropriate translation. The research group based their consensus on which terminology and linguistic concepts were most familiar within the Swedish language and culture without deviating from the original scale. Two independent translators whose mother tongue was English, who speak fluent Swedish, then back-translated the Swedish version of the SEPC-scale. The research group then compared the English native speakers’ translations with both the original English version and the Swedish version. The Swedish version was modified when an essential concept or term was lost in the forward-translation, and if any problematic words or phrases occurred [22, 25].

#### Step 2: expert panel

Six Swedish experts in palliative care were selected as an expert review group (4 men, 2 women). Among the expert group were five Swedish researchers in palliative care; one nurse/professor, one nurse/PhD, two physicians/professors, as well as one nurse/ manager of a competence center for palliative care in Sweden. All participants received written information about the aim of the study and their role as expert reviewers. All six invited experts agreed to participate and returned the questionnaires. The experts assessed all 23-items in the Swedish SEPC-scale on relevance, understandability, clarity, and sensitivity [22, 26]. They ranked each item on a 5-point Likert scale; 1 represented low value and 5 represented high value. The experts also had the opportunity to write comments about each item, make suggestions for other formulations and terms, and offer general observations on the scale. Overall, the returned questionnaires indicated that the experts did not experience difficulties rating the questions. Most of the questions were rated full out by the total number of experts, except for the internal non-responses in questions 3, 4, 8, and 20 in the SEPC-scale (Table 1) where one or two experts left the rating for these questions blank. Nevertheless, in some of these cases, the experts did instead provide an answer in the free text section. Also, with the measure of content validity index, the non-response rate was adjusted.

**Table 1:** SEPC scale relevance: Item, numbers of experts and agreement

Items SEPC-scale (23)	Numbers of experts	Numbers giving a rating of 4–5	I-CVI	Pc^a^	Kappa^b^	Evaluation
***Communication***						
1	6	6	1	0.016	1.00	Excellent
2	6	6	1	0.016	1.00	Excellent
3	5	5	1	0.031	1.00	Excellent
4	4	4	1	0.063	1.00	Excellent
5	6	6	1	0.016	1.00	Excellent
6	6	6	1	0.016	1.00	Excellent
7	6	6	1	0.016	1.00	Excellent
8	5	5	1	0.031	1.00	Excellent
***Patient management***						
9	6	6	1	0.016	1.00	Excellent
10	6	6	1	0.016	1.00	Excellent
11	6	6	1	0.016	1.00	Excellent
12	6	6	1	0.016	1.00	Excellent
13	6	6	1	0.016	1.00	Excellent
14	6	6	1	0.016	1.00	Excellent
15	6	6	1	0.016	1.00	Excellent
16	6	6	1	0.016	1.00	Excellent
***Multidisciplinary teamworking***						
17	6	6	1	0.016	1.00	Excellent
18	6	6	1	0.016	1.00	Excellent
19	6	6	1	0.016	1.00	Excellent
20	5	3	0.60	0.313	0.42	Fair
21	6	5	0.83	0.094	0.81	Excellent
22	6	5	0.83	0.094	0.81	Excellent
23	6	6	1	0.016	1.00	Excellent

*I-CVI* Item-level content validity index

^a^Pc Probability of a chance occurrence. [N!/A!(N-A)!]*.5^ N^. N = number of experts, A = number agreeing on good relevance

^b^Kappa designating agreement on relevance. (I-CVI-pc)/(1-pc). Kappa fair = .40-.59 Good = .60-.74, Excellent > .74

#### Calculation of content validity index (CVI) and modified kappa statistics

One method used to quantify content validity for multiple instruments is to calculate the content validity index (CVI) based on expert ratings of relevance [26]. In this study, the method of CVI-calculation examined the content validity of the Swedish version of the SEPC-scale by calculating CVI on item level (I-CVI). It is common to calculate I-CVI to assess item relevance [26]. When translating and culturally adapting an instrument, it is equally significant to review the instrument for its understandability, clarity, and sensitivity [22]. Therefore, in this study, I-CVI was measured through a calculation within the item level. This was conducted to calculate how the experts valued different parts of an item, in terms of its understandability, clarity, and sensitive aspects, and not only its relevance. A limitation when calculating I-CVI is that it does not answer whether the item’s value depends on change agreement and, consequently, the possibility that the item can get a high value by chance. The kappa statistic, a consensus index of inter-rater agreement that adjusts for chance agreement, has been suggested to measure content validity and complement I-CVI [26]. The present study performed kappa statistics calculations based on the relevance of the items.

When calculating I-CVI, we divided the total number of experts with the number of experts who ranked the item as relevant. To make calculation possible, it was necessary to categorize our Likert scale into two values. Values 1–3 on the Likert-scale were dichotomized to a value of 0 (0 = expert ranked an item low), and the values 4–5 to a value of 1 (1 = expert ranked an item high). The I-CVI expresses the proportion of agreement on the relevancy of each item, which is between 0 and 1 [27, 28]. Furthermore, acceptable content validity is established if an item receives an I-CVI value with a score of < 0.78, using the guidelines by Polit et al. [26]. Their guidelines suggest that an I-CVI higher than 0.78 for three or more experts may be considered confirmation of content validity. Modified kappa statistics (K) are an essential complement to I-CVI and were calculated on item relevance by establishing the I-CVI value and then calculating the probability of chance agreement (PC). The PC-value for each item is computed accordingly: PC = [N!/A! (N -A)!] * 5 N. N = number of experts in a panel and A = number of panelists who agree that the item is relevant. The kappa value was calculated by entering the item’s PC value and the I-CVI value in the following formula: K = (I-CVI—PC) / (1- PC). The cut-off score for kappa was defined as a value of < 0.74 = Excellent [26].

#### Experts’ reviews—a complement to CVI-calculation

The experts’ free answers constituted an essential part of the qualitative review of an item. It created a holistic understanding of the experts’ views about the scale and supplemented I-CVI measurement. The answers were summed and compared based on experts' views of the scales’ relevance, understandability, clarity, and sensitivity [22, 26].

#### Step 3: cognitive interviews

Face-validation considers whether the items appropriately assess the construct in question [29]. The cognitive interview is an essential step in testing the instrument on the target population, with the aim of examining the participant’s experience of the content. The method is commonly used to raise awareness of difficulties or problematic questions that need to be modified to avoid response error [30].

#### Participation and inclusion criterion

In this study, cognitive interview participants were identified and recruited from hospitals (outpatient and inpatient care) and municipal care. The inclusion criteria were individuals over 18 years of age who had experience of working as healthcare professionals. The participants represented a wide range of healthcare professionals (*n* = *10*); physicians s (*n* = *3*), nurses *(n* = *5)* and assistant nurses *(n* = *2)* and included those from both from the north and south of Sweden. The group consisted of three men and seven women, with diverse ages (median 54 years; range 33–57 years). They had a variety of experiences working in palliative care and came from different wards (both hospitals and municipal care). Their time in the profession varied from 11 to 33 years.

#### Performance of interviews, data collection, and data analyses

The performance of ten face-to-face interviews enabled a qualitative analysis of the SEPC-scale. The healthcare professionals received information about the study and its purpose upon their invitation to participate. At the time of the interview, the participants gave their consent and permission to audio record the interview. They also allowed other researchers within the project to take part in the recorded interview and the documentation. Each interview took approximately an hour to complete. Due to the COVID-19 pandemic’s social distancing requirements, seven interviews were conducted through digital meetings via Zoom Video Communications (Zoom), a web-based video conferencing tool [31]. On three occasions, the interviews were conducted in physical meetings at either the participants’ workplace or home.

The cognitive interview procedure employed the “think aloud” method. This method allows the respondents to spontaneously and freely describe (aloud) what they think when they see or hear the question [30]. During the interviews, each item of the SEPC-scale was displayed for the respondents, and they were prompted to describe them aloud. The respondents described what they perceived when they saw an item, repeated the item in their own words, and describe what information they would include when answering the question. They also responded to the questions, whether the statements in the SEPC-scale contained words or phrases that they had positive or negative responses to, and if the statements were considered relevant, understandable, clear, or sensitive [22, 26]. A summary question about the overall experience of the SEPC-scale completed each interview. Two researchers (SA, LG) separately conducted the interviews and performed individual written reports for each interview. Data were summarized and compared based on the respondents’ answers regarding the scales, relevance, understandability, clarity, and sensitivity [22, 26]. All interviews were then reviewed by the research group.

## Results

### Forward and back-translations

Defining anchors on the scale range was a linguistic and adaptation challenge. The anchors “very anxious” to “very confident” were problematic in the Swedish context. In this context, the word “anxious” is not familiar in everyday expressions, nor is it regarded as the opposite of confident. To better fit the Swedish context, the word anxious was changed for a word that was the opposite of confident.

### Relevance

#### Expert panel

The expert panel’s findings showed consensus among the experts that the SEPC-scale was considered relevant. Table 1 shows that 20 of 23 items received an I-CVI value of 1, indicating high content validity. Two other items maintained an I-CVI value over 0.78 and reached a value of acceptable content validation. One item received an I-CVI value under 0.78 and did not reach the accepted value. The excellent kappa values suggest that the experts’ agreement on relevance did not occur by chance. Table 1 also shows the overall response rate and response rate per item (numbers of experts who endorsed a specific item) due to relevance.

#### Cognitive interviews

Healthcare professionals considered that all items on the scale to be relevant to palliative care. However, it is important to note that not all questions were relevant to all professions or wards. For example, the question about pain medication descriptions is only relevant for physicians. There were also different opinions about the degree of relevance in the section on multidisciplinary teamworking. For example, the relevance of lymphoedema service *(item 21)* depended on which profession or ward the participants belonged.

### Understandability, clarity, and sensitivity

#### Expert panel

I-CVI calculation within the item level established that 15 of 23 items reached an approved I-CVI value. Based on the I-CVI calculation, the scale items lack clarity, as shown in Table 2. This was mirrored by the experts, identifying an absence of precision in some items that affected clarity and understanding. The experts also highlighted concerns about the three items that received a low I-CVI value. The expert panel also marked the sentences provide psychological care *(item 14)*, provide social care *(item 15)*, working in a multi-professional palliative care team *(item 17)*, and complementary therapies *(item 20).* The term adequate referring *(items 18–23)* was consistently considered problematic. The experts did not find the scale to be sensitive.

**Table 2:** Calculation of I-CVI on understandability, clarity, and sensitivity

Items SEPC-scale	Understandability		Clarity		Sensitivity	
	I-CVI	Evaluation	I-CVI	Evaluation	I-CVI	Evaluation
1	0.33	Needs revision	0.33	Needs revision	0.83	Approved
2	0.33	Needs revision	0.33	Needs revision	0.83	Approved
3	0.83	Approved	0.67	Needs revision	1	Approved
4	1	Approved	1	Approved	1	Approved
5	1	Approved	1	Approved	1	Approved
6	1	Approved	1	Approved	1	Approved
7	1	Approved	1	Approved	1	Approved
8	1	Approved	1	Approved	1	Approved
9	1	Approved	1	Approved	1	Approved
10	1	Approved	0.83	Approved	1	Approved
11	1	Approved	0.83	Approved	1	Approved
12	1	Approved	0.83	Approved	1	Approved
13	1	Approved	1	Approved	1	Approved
14	0.83	Approved	0.67	Needs revision	1	Approved
15	0.83	Approved	0.50	Needs revision	1	Approved
16	0.83	Approved	1	Approved	1	Approved
17	1	Approved	1	Approved	1	Approved
18	0.83	Approved	0.83	Approved	1	Approved
19	0.83	Approved	0.83	Approved	1	Approved
20	0.60	Needs revision	0.40	Needs revision	0.60	Needs revision
21	1	Approved	1	Approved	0.83	Approved
22	0.83	Approved	1	Approved	1	Approved
23	0.83	Approved	0.67	Needs revision	1	Approved

*Items 1–8: Communication Items 9–16: Patient management Items 17–23: Multidisciplinary teamworking*

#### Cognitive interviews

Healthcare professionals considered the SEPC-scale to be understandable. Difficulties arose when the lack of examples or specificity in items made it unclear how to answer them. In these cases, the items were perceived as too open for interpretation based on the individual’s clinical experience, which gave rise to misunderstandings about what is included in the question. Additionally, they shared the experts’ views on the items provide psychological care *(item 14),* provide social care *(item 15),* working in a multi-professional palliative care team *(item 17),* and complementary therapies *(item 20)*. According to the healthcare professionals, the word provide in these sentences can give rise to misinterpretations in the Swedish context because it can appear unclear who is to perform the act itself; one healthcare professional asked: *shall I give support or refer to support?* As with the experts, several healthcare professionals considered the term adequate referring to be confusing. The items about death and dying woke natural emotions. However, the healthcare professionals did not consider the items insensitive and suggested that the degree of emotional arousal would likely depend on how comfortable the person filling out the questionnaire felt in providing palliative care.

#### The expert panel’s and the healthcare professionals’ overall assessment of the scale

The expert panel review reported that the content of the SEPC-scale covered all important elements in palliative care. Overall, they deemed that the set of items in the SEPC-scale is appropriate for students or healthcare professionals during education, training, or inventions that involve palliative care. They thought that the SEPC-scale can become a useful instrument as the items make students or healthcare professionals reflect on their knowledge in the different areas of palliative care. The expert panel, however, did raise some concerns regarding the precision of items in multidisciplinary teamworking.

## Final version

### Changes made due to the evaluation of the relevance

After step 2 (expert panel), the word “cancer” was excluded and replaced with the word “illness” *(items 1–2)*. After step 3 (cognitive interviewing), the response option *not applicable* was added to the item about pain medication descriptions *(item 12)* as an alternative for other professions. The item about lymphoedema service *(item 21*) was excluded and replaced with somatic care. Additionally, a new item was added that mentioned specialist palliative care service. To summarize, these changes were made with the intent to make items in the instrument more relevant for those who care for seriously ill patients with non-oncological illnesses, and more pertinent for professions other than medical students and physicians.

### Changes made due to the evaluation of the understandability, clarity, and sensitivity

After step 2, minor modifications in the form of linguistic changes were made to clarify items in the SEPC-scale. After step 3, the following changes were made: working in a multi-professional palliative care team *(item 17)* was changed to another formulation about team working in palliative care. The item about complementary therapies *(item 20)* was provided with examples (acupuncture, tactile treatment/massage), to clarify what form of treatments could be included in the Swedish context. In the sentences provide psychological care *(item 14)* and provide social care *(item 15),* the word provide was changed from its previous Swedish translation in step 1 (forward and back-translation) (*erbjuda*) to the word (*ge*) because it increases the clarity based on Swedish expressions. In addition, after step 3, the term appropriately referring was extended to include the word identify; appropriately identify and if necessary, refer patients in need of palliative care to… The intention was to clarify these items. Healthcare professionals may not always talk about referring because it is a technical term in the Swedish healthcare setting, and therefore excludes other ways to identify and inform other professionals about a patient’s need. Additionally, not all professionals have the right to refer patients; for example, assistant nurses may find that these items not relevant to their profession. Table 3 below shows the essential modifications of the items in the SEPC-scale. Items most commonly needed modification in the section on multidisciplinary teamworking.

**Table 3:** Modification of items in SEPC-scale, step 1–3

Items SEPC-scale (original)	Modifications: Step 1: Forward-back translation	Modifications: Step 2: Expert panel	Modifications: Step 3: Cognitive interviews
**Communication** (1) discussing the likely effects of cancer with the patient (2) discussing the likely effects of cancer with the patient’s family	(1) discussing the likely effects of cancer/illness with the patient (2) discussing the likely effects of cancer/illness with the patient’s family	(1) discussing the likely consequences of the illness with the patient (2) discussing the likely consequences of the illness with the patient’s family	No modifications
**Patient management** (12) your ability to prescribe appropriate and adequate pain control medication	No modifications	No modifications	(12) your ability to prescribe appropriate and adequate pain control medication* not applicable
**Multidisciplinary teamworking** (17) working within a multi-professional palliative care team	No modifications	No modifications	(17) Working in a team with different professions in palliative care
(18) appropriately referring palliative care patients for physiotherapy (19) appropriately referring palliative care patients for occupational therapy (20) appropriately referring palliative care patients for complementary therapies	No modifications	No modifications	(18) appropriately identify and if necessary, referring patients in need of palliative care to physiotherapy (19) appropriately identify and if necessary, referring patients in need of palliative care to occupational therapy (20) appropriately identify and if necessary, referring patients in need of palliative care to complementary therapies (acupuncture, tactile treatment/massage)
(21) appropriately referring palliative care patients to a lymphoedema service	No modifications	No modifications	(21) appropriately identify and if necessary, referring patients in need of palliative care to somatic assessment (22) appropriately identify and if necessary, referring patients in need of palliative care to specialist palliative care services

## Discussion

The consensus among experts and healthcare professionals about the relevance of the SEPC-scale confirms that the instrument is considered applicable and usable in the Swedish palliative care context. Reviews by healthcare professionals showed that the scale could be usable for students during education, and that it could be an important tool allowing healthcare professionals to reflect on their performance in palliative care.

The aim of this study is to provide the Swedish healthcare settings with a broad instrument aimed at contexts that both include and go beyond cancer, and for professions other than medical students and physicians, with the goal to include other diagnostic groups with palliative care needs. For this purpose, items that were perceived to only be relevant to a certain profession, diagnosis, or clinical setting required linguistic or contextual modifications. The original SEPC-scale is, to some extent, focused on cancer diagnoses. The specification of cancer excludes other patient groups with palliative care needs. In the Spanish version of the SEPC-scale the word “cancer” was replaced with “illness” [19]. This was also carried out in this study, since it is more equivalent to the World Health Organization’s view of palliative care and its fundamental values [1]. To strengthen relevance for a wider range of professions and clinical settings, the focus on lymphoedema service was excluded and exchanged for two items that were considered more relevant for different care settings. In the Spanish version this item was also excluded and replaced [19]. The Swedish version of the SEPC-scale received some negative reviews due to its understanding and clarity. An item could be valued as highly relevant but receive a lower grade regarding the linguistic wording. Previous translation studies refer to this problem; translation can involve a linguistic challenge because two languages can have non-equivalent words or idiomatic expressions, but also language and culture are two basic features according to cross-culture studies when adapting instruments from one culture to another [25, 32]. In this study, it was necessary to change formulations and to exclude or add words and explanations so that the instrument was more acceptable to those who were administering it. Culture and language are intimately linked [32], as demonstrated in the current study. This study also showed that the questions in this scale about death and dying evoke emotions, but not in a negative way. The healthcare professionals argued that the ability to reflect on death and dying is a key foundation within palliative care, and that health care professionals must consider these issues and develop their self-confidence to approach them. They also stated that the SEPC-scale does not arouse sensitive emotions in those confident with these issues, but insecure healthcare professionals may perceive the items as more challenging. Similarly, a study by Gryschek et al. [20] showed that being uncomfortable with death and dying creates more anxiety and less confidence to approach dying patients, indicating that the higher the fear of death, the lower the self-efficacy in palliative care. Previous healthcare research has shown that the theoretical foundation of self-efficacy can be appropriate when evaluating palliative care confidence [33, 34]. Regardless, it is noteworthy to mention that self-efficacy is not a measure of someone’s capability [12]; as Bandura describes, self-efficacy is only *one* factor according to the framework of social cognitive theory along with others that can influence action and behavior [35]. The SEPC-scale is, therefore, not suitable to measure competencies in palliative care, but it can provide information regarding what abilities health care professionals *believe* they possess.

### Methodological considerations

The main strength of this study is the robust methodological mixed-method design, which involved collecting both qualitative and quantitative data from experts in palliative care and from healthcare professionals. This permitted a more thorough cross-cultural adaptation, including a systematic methodological description of the translation process, adaptation, and a validation study. However, the translation and adaptation process required a balancing act to modify language and adjust items while maintaining the purpose of the scale. This was particularly challenging in this study since it aimed to broaden the perspective of the original SEPC-scale. The expert panel and cognitive interviews showed the importance of considering both content validity and face-validity [29] in the Swedish context. It was common that an item received a high value regarding its relevance but was graded lower in understandability and clarity. This rating confirms the significance of an overall assessment of an item, including understandability, clarity, and sensitivity, and means that relevance should not be exclusively relied upon. The SEPC-scale was validated through both quantitative (content validity) and qualitative (cognitive interviewing) methods. Completing the CVI and kappa statistics calculations were essential steps for the early process of evaluation of an instrument [26]. Validation requires thorough work, as emphasized, hence, several studies may be required to adequately describe the whole validation process [36]. Still, a limitation of this study is that the Swedish version of the SEPC-scale has not been tested on a larger population of healthcare professionals, which would allow us to analyze the outcome of the scale. However, this study is the first stage in a continuing process. The next step will be to investigate the SEPC-scale further through psychometric analyses, for instance, factor analysis. In further studies, the SEPC-scale will be applied to include a larger sample of healthcare professionals (for example physicians, nurses, and assistant nurses) in clinical settings to measure their confidence in palliative care.

## Conclusion

The study showed that the Swedish version of the SEPC-scale is applicable to healthcare professionals in Swedish settings and assesses both their perceived capability to deliver palliative care and their emotional self-confidence to cope with situations that refer to death and dying. Following the core values of palliative care, this study also took the opportunity to construct a broader version of the instrument by modifying the items to include various healthcare professions, and multiple care contexts and diagnoses. Cultural adaptation when translating an instrument is vital, establishing relevance and enabling acceptance to various healthcare professionals and contexts. Using the methodological approach of an expert panel and cognitive interviewing, the SEPC-scale is considered valid for use in Swedish healthcare practice, for multiple care settings and healthcare professionals, and is not solely specific to cancer care or medical students and physicians.

## Data Availability

Data are available from the corresponding author upon reasonable request.
